# Indigenous Culture and Health in UDRH Research: An Indigenous‐Led Narrative Review

**DOI:** 10.1111/ajr.70175

**Published:** 2026-04-13

**Authors:** Michael Watkins, Colleen Kelly, Charmaine Green, Samantha Bay, Ginger Minahan, Bahram Sangelaji, Sandra C. Thompson

**Affiliations:** ^1^ Department of Rural Health Adelaide University Whyalla Norrie South Australia Australia; ^2^ IIMPACT in Health Adelaide University Adelaide South Australia Australia; ^3^ Health and Social Care Unit Monash University Clayton Victoria Australia; ^4^ Western Australian Centre for Rural Health University of Western Australia Geraldton Western Australia Australia; ^5^ Columbia University Mailman School of Public Health New York New York USA; ^6^ Southern Queensland Rural Health Cranley Queensland Australia

**Keywords:** Aboriginal and Torres Strait Islander, culture, Indigenous, rural health

## Abstract

Whilst University Departments of Rural Health (UDRHs) have undertaken research relevant to Indigenous health over decades, understanding of the contributions in the context of culture and health has been largely unexplored.

**Objective:**

Through use of culturally appropriate methodology, this Indigenous‐led narrative review aimed to summarise how the research has responded to Indigenous perspectives and priorities to provide insights into how UDRH research has considered Indigenous cultural ways, and summarise benefits associated with UDRH studies.

**Design:**

This narrative review analysed published UDRH Indigenous health research using systematic analysis and collaborative yarning.

**Results:**

Thirty‐three papers were reviewed. Key themes identified Indigenous involvement and governance, self‐determined research, Indigenous research methodologies, culture as a determinant of health and Indigenous perspectives and understanding of health phenomena, which featured across the included studies.

**Conclusions:**

UDRH Indigenous culture and health research studies highlight Indigenous participation and governance in research processes which contribute to positive health and well‐being outcomes and build research capacities of both Indigenous and non‐Indigenous researchers.

## Keypoints

**What This Paper Adds:**

- University Departments of Rural Health (UDRHs) have engaged in decades of research with Indigenous people. This Indigenous‐led narrative review examines a subset of UDRH Indigenous health research to provide insights into how UDRHs have specifically considered Indigenous culture in their research into Indigenous health.
- It examines how they have responded to Indigenous cultural perspectives and priorities in their health research with Indigenous people.

**What is Already Known on This Subject:**

- UDRHs have a long history of engaging in health research with Indigenous Australians as part of their efforts to improve Indigenous health.
- There is a critical need for Indigenous‐led, co‐designed and community‐initiated research that embodies Indigenous ways of knowing, being and doing to address research priorities.

Existing research related to Aboriginal and Torres Strait Islander (hereafter respectfully referred to as Indigenous) peoples is often framed in terms of health disparities, and even more so for people living in rural and remote regions, problematising individuals and communities. Indigenous peoples are questioning colonising research methods associated with a history of being over researched without significant health improvements [1, 2, 3].

Reviews of Australian Indigenous health research found that biological or clinical models of health dominate the peer‐reviewed literature, with a lack of Indigenous health models [4]. Research has often employed methodologies that do not align with Indigenous ways of knowing, being and doing, and resulting in efforts to provide solutions are culturally misaligned and euro‐centric [5]. Indigenous concepts of health and well‐being are considered holistic, expanding beyond Western concepts, encompassing interconnected social and cultural knowledge [2]. While it is possible to conduct culturally informed research through the use of both Indigenous and non‐Indigenous methods, it is increasingly recognised that efforts to improve Indigenous health must prioritise Indigenous priorities, practices, values and knowledge [5]. Researchers should consider holistic approaches attending to Indigenous knowledge and cultural practices in study designs [6].

There is also recognition of the important role academic institutions can play in building capacity of Indigenous researchers, ensuring ethical practices and their role in addressing Indigenous health disparities [3, 7]. University Departments of Rural Health (UDRHs) were first established more than 25 years ago through national funding to enhance rural health workforce capacity and are important providers of health service delivery and research in rural and remote areas of Australia [8]. The Australian Rural Health and Education Network (ARHEN) is a national association for the 19 UDRHs which links isolated academic centres to other UDRHs and provides opportunities for clinical educators, health professionals and researchers to learn from and collaborate with each other. UDRHs have significant involvement with Indigenous people in the regions in which they work. Appointment of Indigenous staff and projects with local Indigenous people and organisations have been an important part of the work of UDRHs. ARHEN has supported Indigenous staff from UDRHs around Australia to participate in a longstanding staff network, the Aboriginal and Torres Strait Islander Staff Alliance (ATSISA), providing an opportunity for Indigenous academic, professional and support staff to connect and collaborate with colleagues.

While UDRHs have undertaken considerable research relevant to Indigenous health over decades, understanding these contributions in rural and remote contexts has been largely unexplored. UDRH researchers develop evidence on rural and remote health issues and advocate for these locally, regionally and nationally. Indigenous UDRH staff contribute to the cultural capital of rural and remote health through Indigenous‐led research, working with health services and building research capabilities of local workforce and UDRH staff [9, 10]. This Indigenous‐led review aimed to examine and summarise the significant historical contributions of UDRHs to Indigenous health research from a national rural network committed to building Indigenous research capacity and identifies opportunities to improve future Indigenous health research practices to which UDRH's contribute.

## 1 Methods

In 2022, ARHEN established a database of peer‐reviewed papers from 2010 on which UDRH were contributing authors. Collaborators from multiple UDRHs summarised the nature of Indigenous health research from ARHEN's database published between 2010 and 2021 [11]. Interest in the contributions of UDRHs to Indigenous research led to a proposal to explore this substantial body of research in more depth. In the initial analysis, Thompson et al. [11] grouped the 493 Indigenous focussed publications into categories including ‘health services research’, ‘epidemiology’ and ‘Indigenous culture and health’. This narrative review forms part of a broader project of more in‐depth exploration of individual research categories based on the initial examination of Indigenous health research from ARHEN's 2010–2021 database.

Following the ARHEN Board's approval, the proposal of this study was discussed with members of the ATSISA, with members invited to contribute if they had interest and capacity to ensure an Indigenous cultural lens and that the methods aligned with Indigenous ways of knowing, being and doing. Indigenous authors yarned about aligning this review with Indigenous ways of knowing, being and doing. It was agreed that a narrative review led by Indigenous authors would be the most appropriate way to explore the papers.

To our knowledge, there are currently no published guidelines on how to conduct culturally appropriate narrative reviews. Authors of this narrative review incorporated Indigenous Research Methodologies (IRM) drawing on the work of Brodie et al. [5] which centres Indigenous worldviews and values throughout the review process. This approach includes a team of Indigenous and non‐Indigenous researchers with varied experience in both Indigenous and western research methodologies. The inclusion of Indigenous perspectives in the analysis of the literature and results reflects a prioritisation of Indigenous ways of knowing, being and doing through our process and methods, enhancing a traditional narrative review method by incorporating an Indigenous research approach [5, 12].

### 1.1 Inclusion/Exclusion Criteria

The papers reviewed in this article were sourced from ARHEN's database of publications to which UDRH researchers contributed between 2010 and 2021. The 40 publications from the category ‘Indigenous culture and health’ in Thompson et al. [11] paper were examined in this narrative review. Studies classified in this category explored cultural values, delivery of services in a culturally safe way, and barriers to health access for Indigenous people due to insufficient attention to cultural values [11]. This narrative review was conducted concurrently with other reviews of the categories identified in Thompson et al. [11] paper (e.g., ‘workforce’ and ‘intervention’ papers). Our focus was on studies undertaken rather than those planned, so protocol papers were excluded.

### 1.2 Data Extraction

We used the Australian Indigenous HealthInfoNet ‘Cultural Ways’ [13] as a framework for data extraction to conceptualise research related to Indigenous culture and health. ‘Cultural Ways’ highlights six important domains that contribute to culturally safe research and programmes for Indigenous peoples identifying (1) Aboriginal and Torres Strait Islander concept of health, (2) traditional healing and medicine, (3) cultural practices, (4) identity, (5) working with community and (6) data sovereignty.

Data extraction was completed in two stages. Firstly, authors independently examined the papers and summarised each study in a data extraction spreadsheet: state of study site/geographical context, which domain(s) of ‘Cultural Ways’ were considered, the aims, participants, methodology, key findings, and recommendations. All papers were coded by at least two authors, with an additional author consulted to resolve any discrepancy.

Authors then extracted additional information about how each study had considered the domain of ‘Cultural Ways’ information about why each study was conducted, the involvement of Indigenous people in the studies, period of engagement with participants, outcomes of the studies, who the studies benefited and if there was evidence of providing feedback about outcomes to Indigenous communities.

### 1.3 Data Analysis

Authors analysed the data through an iterative, Collaborative Yarning process [4, 14, 15]. Collaborative Yarning is an IRM that extends beyond the broader method of yarning which is underpinned by Indigenous values of relationality, responsibility and cultural accountability [16]. Collaborative Yarning was conducted organically through creating a shared space for storytelling, reflection and meaning making based in trust, reciprocity and cultural safety, and considering all parties as knowledge holders in the research process [17].

Collaborative yarning methodology honours Indigenous values and privileges Indigenous perspectives in the research process [15]. Initially, each Indigenous team member reviewed the data extracted in the spreadsheets independently, then engaged in a series of collaborative yarns undertaken online to accommodate the authors' geographical separation. The authors recognise the interconnectedness of themes when constructing knowledge while adhering to the methodological parameters of a narrative review, recognising that these parameters may limit the extent to which Indigenous health and culture can be represented [18].

Collaborative yarning enabled themes to be iteratively co‐created and identified collaboratively. Following this, non‐Indigenous authors were included in discussions about the themes that emerged from the collaborative yarning process to further refine and synthesise the results. This method of reviewing papers individually and collectively as a research group allowed opportunity to consider the interconnected concepts of culture, health and well‐being [6].

## 2 Results

**FIGURE 1:**
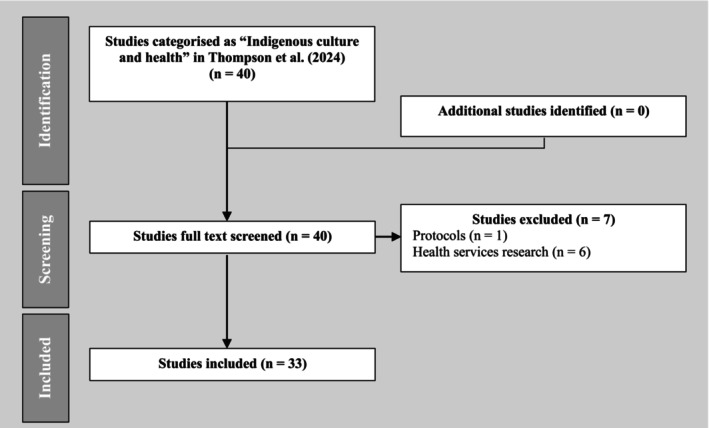
Flow chart of inclusion and exclusion process.

In the original classification of Indigenous research from ARHEN's database [11], 40 papers were categorised by the authors in the ‘Indigenous culture and health’ category. After reading each paper, with reference to the descriptions of ‘Cultural Ways’ from the Australian Indigenous HealthInfoNet, and discussions among the authors, seven papers in total were excluded from the analysis. Six of the seven papers excluded were reclassified as health services research, as their focus was on health service improvement. An additional paper was excluded as it was a study protocol. This left 33 papers for analysis in this narrative review. Figure 1 summarises the inclusion and exclusion process.

Data extracted from the 33 studies are presented in Tables 1 and 2. Table 1 summarises for each paper the study designs and engagement with Indigenous people, while Table 2 summarises the insights on Indigenous health and culture, and benefits associated with studies.

**TABLE 1:** Summary of study designs.

References, title	States, rurality of study site	How the research arose	Indigenous people's involvement	Participants	Period of engagement with Indigenous people	Method of data collection	Feedback of findings to the community
Nichols [19], Aboriginal recommendations for substance use intervention programs	WA, remote	Requested by the community due to ineffectiveness of current programmes	Participatory action design; research was requested by local Indigenous people	Indigenous adults	Not reported	Semi‐structured interviews and focus groups	Not reported
Schoen et al. [20], Health promotion resources for Aboriginal people: lessons learned from consultation and evaluation of diabetes foot care resources	WA, urban, rural and remote	High rate of amputation due to diabetes	Aboriginal reference group; Indigenous participants chose/created resources	Indigenous adults	Not reported	Focus groups	Not reported; but community members were involved in creation of resources
Shahid et al. [21], ‘If you don't believe it, it won't help you’: use of bush medicine in treating cancer among Aboriginal people in Western Australia	WA, urban, rural and remote	Gap in literature about traditional healing	Aboriginal reference group; Aboriginal co‐authorship	Indigenous adults	Unspecified period of engagement with community; 6‐ month data collection period	Interviews, observations and field notes	Not reported
Coffin [22], ‘Make Them Stop it’: What Aboriginal Children and Youth in Australia Are Saying About Bullying	WA, rural and remote	High prevalence of bullying, gap in the literature about bullying in Aboriginal children and adolescents	Aboriginal author, Aboriginal research assistants, Aboriginal steering group	Indigenous children and youth	3 years	Interviews	Validation from communities sought; summary for each region; newspaper articles; conference presentations
McBain‐Rigg and Veitch [23], Cultural barriers to health care for Aboriginal and Torres Strait Islanders in Mount Isa	QLD, rural	Inconsistencies in definition of ‘cultural barriers’	Aboriginal culture mentors	Indigenous and non‐Indigenous healthcare workers	2 years	Semi‐structured Interviews	Not reported
Passey et al. [24], ‘Its almost expected’: rural Australian Aboriginal women's reflections on smoking initiation and maintenance: a qualitative study	NSW, rural	High smoking rates in Indigenous Australians, gap in literature about initiation of smoking in rural Indigenous women	Elders council, community reference group	Indigenous women and service providers	Not reported	Focus groups and interviews	Results presented to the community reference group
Lin et al. [25], ‘I am absolutely shattered’: the impact of chronic low back pain on Australian Aboriginal people	WA, rural and remote	Paradoxical findings (low levels of disability vs. high levels of prevalence of chronic disease) in previous studies	11 Aboriginal community leaders	Indigenous adults	Period of engagement with community leaders unspecified; Data collection 3 years	Interviews (yarning)	Not reported
Taylor et al. [26], Intercultural communications in remote Aboriginal Australian communities: What works in dementia education and management?	NT, remote	High incidence of dementia in remote Indigenous populations	Indigenous advisory group; Indigenous researcher	Indigenous aged care workers, Indigenous adults, non‐Indigenous staff	Not reported	Focus groups, interviews and field notes	Not reported
Lin et al. [27], Disabling chronic low back pain as an iatrogenic disorder: a qualitative study in Aboriginal Australians	WA, rural and remote	Gap in literature about beliefs about chronic lower back pain and the relationship to disability	11 Aboriginal community leaders	Indigenous adults	Period of engagement with community leaders unspecified; Data collection 3 years	Interviews (yarning)	Not reported
Wild et al. [28], ‘Give us the full story’: overcoming the challenges to achieving informed choice about fetal anomaly screening in Australian Aboriginal communities	NT, urban and remote	Lower screening rates in Indigenous women compared to non‐Indigenous women	Indigenous reference group, Indigenous researcher	Healthcare providers, Indigenous women	Not reported; once‐off interviews with participants	Interviews, group discussions and field notes	Findings forwarded to organisations, participants received an email
Cairney and Abbott [29], Aboriginal well‐being in a ‘Red Dirt Economy’	NT, SA and WA, remote	Gap in current government policy and services in maintaining integrity of values and worldviews across cultural, political and scientific interfaces	Aboriginal ownership across various levels. 50% Indigenous representation in management, Indigenous team members, Indigenous community researchers	Indigenous adults (researchers, leaders, elders)	7 years of funding; 3 years data collection	Workshops and interviews	Communities were engaged throughout the study
Jurgenson et al. [30], Exploring Australian Aboriginal women's experiences of menopause: a descriptive study	WA, rural	Gap in literature about Indigenous women's experiences of menopause	Indigenous co‐author	Indigenous women	1 year	Interviews and focus groups	Not reported
Lin et al. [31], ‘I can sit and talk to her’: Aboriginal people, chronic low back pain and healthcare practitioner communication	WA, rural and remote	Poor communication practices despite healthcare professionals' good intentions	Indigenous co‐investigators	Indigenous adults	Period of engagement with community leaders unspecified; Data collection 3 years	Interviews (yarning)	Not reported
Lopes et al. [32], Cross cultural education in suicide prevention: Development of a training resource for use in Central Australian Indigenous communities	NT, Rural	High suicide rates in NT, few mental health services available to meet needs of Indigenous people	Indigenous advisor, Indigenous interpreters, Indigenous researchers	Workshop trainees (healthcare workers and community members); five were Indigenous	Not reported	Interviews	Not reported
Rae et al. [33], Long conversations: Gomeroi gaaynggal tackles renal disease in the Indigenous community	NSW, rural and remote	High rates of end stage renal disease in Indigenous populations	Programme facilitated by Indigenous Elder and artist, ‘in partnership with Indigenous communities’	Young Indigenous women	Not reported; study ongoing since 2009	Samples collected during pregnancy for analysis of biomarkers, self‐report on smoking	Not reported
Rix et al. [34], ‘Beats the alternative but it messes up your life’: Aboriginal people's experience of haemodialysis in rural Australia	NSW, rural	Gap in literature about experiences of rural Indigenous Australians undergoing haemodialysis	Indigenous community reference group (patients, Elders, health workers), Indigenous researchers; Indigenist and Community Based Participatory Research (CBPR)	Indigenous adults	Period of community engagement not reported; data collection 8 months	Interviews (yarning)	Feedback provided to health professionals; not reported for other stakeholders
Zander et al. [35], Indigenous Cultural and Natural Resources Management and Mobility in Arnhem Land, Northern Australia	NT, remote	Challenges to providing services to mobile Indigenous people; gap in literature about relationships between CNRM and mobility	Indigenous co‐researchers	Indigenous adults	Data collection 13 months	Interviews	Not reported
Cuesta‐Briand et al. [36], Addressing unresolved tensions to build effective partnerships: lessons from an Aboriginal cancer support network	WA, rural	Gap in literature about the function and effectiveness of Indigenous‐specific cancer peer‐support programmes	Not reported, but this study was part of a larger project	Indigenous healthcare workers, Indigenous members and clients	Not reported	Interviews, workshop forum	Not reported
Gladman et al. [37], Measuring organisational‐level Aboriginal cultural climate to tailor cultural safety strategies	Not reported, rural	RCS made efforts to increase cultural safety; this study was an evaluation	Aboriginal‐led study	RCS staff (clinical, academic, professional)	Not reported	Online survey	Not reported
Helps and Barclay [38], Aboriginal women in rural Australia; a small study of infant feeding behaviour	NSW, rural	Low breast‐feeding rates in rural Aboriginal mothers, having impact on infant health and long‐term outcomes	Cycles of collaboration with participants, informants and mentors. Aboriginal staff commented on the summary of the study.	Aboriginal women	Not reported	Semi‐ structure interviews	Knowledge translation through variety of engagement approaches and outputs
Cairney et al. [39], Interplay well‐being framework: a collaborative methodology ‘bringing together stories and numbers’ to quantify Aboriginal cultural values in remote Australia	NT and WA, remote	Lack of culturally and scientifically validated framework of Indigenous Australian well‐being	Participatory action research approach which engaged end users and Aboriginal reference group	Aboriginal peoples in remote Australia	2014–2015 data collection	Survey data	Knowledge translation through various engagement approaches and outputs
McPhail‐Bell et al. [40], Deadly Choices empowering Indigenous Australians through social networking sites	Not reported (Online)	Gap in literature about effective social network sites for community‐based health promotion for Indigenous Australians	Programme designed by Aboriginal peoples of SE QLD	Aboriginal participants of Deadly Choices (unknown demographics)	2 years (online)	Ethnographic interviews, conversations and online fieldwork	Community actively engaged throughout study, feedback to community was not reported
Pilkington et al. [41], Perspectives of Aboriginal women on participation in mammographic screening: a step towards improving services	WA, urban and rural	Low participation of Indigenous women in breast screening despite proactive agency efforts	Aboriginal researchers and Elders involved and consultation, including Aboriginal reference group	Aboriginal women and Aboriginal health professionals	Not reported	Semi‐structured interviews, yarning and focus groups	Not reported
Vujcich et al. [42], Yarning quiet ways: Aboriginal carers' views on talking to youth about sexuality and relationships	WA, urban and rural	Previous findings that Indigenous youth wanted parents and Elders to play an active role in sexual education; recommendations by the Metropolitan Sexual Health Action Group	Indigenous reference group and Indigenous organisations consulted	Indigenous adults (carers)	Not reported	Focus group, semi‐structured interviews	Not reported
Schultz et al. [43], Indigenous land management as primary health care: qualitative analysis from the Interplay research project in remote Australia	NT and WA, remote	Requested by Indigenous community leaders	Indigenous authors, Indigenous community researchers	Indigenous and non‐Indigenous service providers and users	2014–2015	Focus groups, interviews	Not reported
Schultz et al. [44], Injury prevention through employment as a priority for well‐being among Aboriginal people in remote Australia	NT and WA, remote	Injury is a common cause of death, and significantly impacts quality of life in Indigenous people	Aboriginal community researchers employed	Aboriginal and non‐Aboriginal participants	2014–2015	Focus groups, interviews	Not reported
Schultz et al. [45], Re‐Imagining Indigenous Education for Health, Well‐being and Sustainable Development in Remote Australia	NT and WA, remote	Indigenous communities and government concerned about poor education outcomes of Indigenous children in remote regions	Indigenous community researchers employed	Indigenous and non‐Indigenous participants	Not reported	Focus groups	Not reported
Armstrong et al. [46], ‘I've got to row the boat on my own, more or less’: Aboriginal Australian experiences of traumatic brain injury	WA, rural and remote	Gap in literature about traumatic brain injury in Indigenous people	Aboriginal interviewers who were trained for the study	Aboriginal men	Not reported	Interviews	Not reported
Dew et al. [47], Importance of Land, family and culture for a good life: Remote Aboriginal people with disability and carers	NT, SA and WA, remote	Challenges in accessing appropriate services for disabled Indigenous people; and gap in literature about Indigenous perspectives on disability	Senior Aboriginal women provided cultural context to research results, Aboriginal interviewers	Aboriginal peoples with disability, family carers and non‐Aboriginal workers	Not reported	Interviews	Visual depiction of conceptualisation of research by senior Anangu woman, dissemination methods not detailed in paper.
Schultz et al. [48], Australian Indigenous Land Management, Ecological Knowledge and Languages for Conservation	NT and WA, remote	Challenging the common representations of Indigenous Australians; highlighting Indigenous cultural, ecological and language knowledge	Study tool developed and administered by Indigenous researchers	Indigenous and non‐Indigenous participants	Not reported	Survey	Unclear
Schultz et al. [49], Structural modelling of well‐being for Indigenous Australians: importance of mental health	NT and WA, Remote	Biomedical frameworks lack reference to Indigenous people's well‐being priorities	Indigenous leadership and governance. Indigenous researchers develop and administered study tool	Indigenous adults	12 months	Survey	No reported
Thompson et al. [50], Passing on wisdom: exploring the end‐of‐life wishes of Aboriginal people from the Midwest of Western Australia	WA, Rural	Underutilisation of palliative care by Indigenous people and poor availability of culturally safe palliative care services	Aboriginal researcher undertook community engagement	Aboriginal adults	Not reported	Group discussions	Community engaged throughout, videos created for educational purposes
Cox et al. [51], ‘It all comes back to community!’: A qualitative study of Aboriginal Elders promoting cultural well‐being	WA, Remote	Gap in literature about Elders' contributions to Indigenous people's well‐being	Co‐design led by community Elder and Aboriginal authors	Aboriginal elders	3 months	Yarning circles	Not reported

**TABLE 2:** Insights to Indigenous concepts of health and culture.

References, title	‘Cultural Ways’ domains considered (Aboriginal and Torres Strait Islander concept of health; Traditional healing and medicine; Cultural practices; Identity; Working with community; Data governance and sovereignty)	Insights to Indigenous health and culture	Benefits of the study
Nichols [19], Aboriginal recommendations for substance use intervention programs	Concept of health; Cultural practices; Identity	Concept of health: Explored opinions and experiences of services in context of cultural and social needs, self‐determination and skills Cultural practices: Participants determined AOD programmes to be ineffective due to not taking cultural ways of knowing, being and doing into account in design or execution Identity: Educational, personal, family and support recommendations of programme evaluations demonstrated priorities for a focus on vocational, educational, identity‐ and family‐related intervention components	Indigenous Research Reform Agenda recommendations to improve future interventions. Highlighted the importance of physical and emotional support, substance use and life skills education, and socialising aspects of interventions. Identified need to change fundamental skills for self‐determination, cultural safety and local services for rural areas.
Schoen et al. [20], Health promotion resources for Aboriginal people: lessons learned from consultation and evaluation of diabetes foot care resources	Concept of health; Cultural practices; Working with community	Concept of health: Explored preferences of materials and delivery that would appeal to Aboriginal peoples' concept of health Cultural practices: Traditional art, language and stories used in resources as way to impart health knowledge Working with community: Aboriginal participants determined and endorsed relevant health promotion resources	Self‐determined community approach for delivery of health promotion and education about diabetic foot care, with recommendations for future initiatives
Shahid et al. [21], ‘If you don't believe it, it won't help you’: use of bush medicine in treating cancer among Aboriginal people in Western Australia	Traditional healing and medicine; Cultural practices	Traditional healing and medicine: Study explores contemporary meanings attached to the use of bush medicine Cultural practices: Explored the wishes of Aboriginal people to use bush medicine alongside mainstream cancer treatment	Led to development of an Indigenous women's cancer support group. Highlighted Aboriginal people's desire to use traditional medicine associated with a holistic view of health
Coffin [22], ‘Make Them Stop it’: What Aboriginal Children and Youth in Australia Are Saying About Bullying	Concept of health; Identity; Working with community; Data governance and sovereignty	Concept of health: Youth identified life in community, family and community connectedness, school life, home life and supportive individuals in context of well‐being Identity: Explores and establishes Aboriginality and identity and need to build strong Aboriginal identity for Aboriginal youth to maintain well‐being Working with community: Need for locally developed and informed, culturally secure practice to support Aboriginal students Data governance and sovereignty: Tailored summaries for different regional groups provided	Capacity building of Aboriginal research assistants and empowered youth's voices in research. Recommendations provided were culturally, linguistically and geographically specific. Recommendations for interventions to provide staff training, psychological support and locally developed resources about bullying.
McBain‐Rigg and Veitch [23], Cultural barriers to health care for Aboriginal and Torres Strait Islanders in Mount Isa	Working with community	Cultural practices: Culture misinterpreted as becoming cultural barriers rather than culture informing ways of engaging with each other and systems Working with community: The design of the study considered cultural sensitivities, including gender sensitivities and implemented separate male and female groups which allowed for cultural safety.	Highlighted barriers and made recommendations to improve cultural safety in health services.
Passey et al. [24], ‘Its almost expected’: rural Australian Aboriginal women's reflections on smoking initiation and maintenance: a qualitative study	Identity; Working with community	Identity: Young girls use smoking to assert Aboriginal identity and group membership as a way of belonging; importance of Aboriginal identity and culture as source of strength and empowerment Working with community: Community involvement is necessary for outcomes regarding smoking among Aboriginal people	Acknowledged Aboriginal identity and culture as a source of empowerment. Provided insights into factors associated with smoking, and recommendations for strategies to reduce uptake of smoking and increase smoking cessation in Aboriginal females.
Lin et al. [25], ‘I am absolutely shattered’: the impact of chronic low back pain on Australian Aboriginal people	Concept of health; Working with community	Concept of health: Explored socio‐cultural influences on CLBP disability including protected factors Working with community: Drawing attention to culturally secure approaches, such as visual formats, understandable language and supportive future use of the approach in practice	The study's findings challenge the assumption that Aboriginal people are buffered from CLBP disability, and advocated for more appropriate attention.
Taylor et al. [26], Intercultural communications in remote Aboriginal Australian communities: What works in dementia education and management?	Concept of health; Cultural practices; Identity	Concept of health: Participants expressed themes around connection to culture, awareness of symptoms, impact of good communication with family and communities Cultural practices: Explored the impact of intercultural communication; Aboriginal peoples and use of Aboriginal languages Identity: Use of Aboriginal languages in resources as culturally safe intercultural communication	Identified components of successful inter‐cultural communication. Increased awareness and understanding about dementia that led to noticeable behavioural and attitudinal change.
Lin et al. [27], Disabling chronic low back pain as an iatrogenic disorder: a qualitative study in Aboriginal Australians	Concept of health; Cultural practices; Working with community	Concept of health: Potential erosion of traditional cultural concepts of health if continued exposure to biomedically orientated approaches for CLBP Cultural practices: Research established theoretical framework to validate and understand experiences of Aboriginal participants Working with community: Communicate in ways that builds positive beliefs about back pain and enhances resilience	Highlighted beliefs of Aboriginal people, about CLBP and relationship to disability. Advocated for culturally informed care.
Wild et al. [28], ‘Give us the full story’: overcoming the challenges to achieving informed choice about fetal anomaly screening in Australian Aboriginal communities	Concept of health; Working with community	Concept of health: Explored women's perceptions on improved communication and examined cultural complexities on screening consideration socio‐cultural context Working with community: Interviews showed Aboriginal women wanted communication processes to include discussions led by Elders and educators; promote culturally defined ways of sharing information rather than individualised, biomedical approaches in clinical setting	Highlighted barriers to accessing fetal anomaly screening, and study advocates that clinicians make more effort to ensure that they offer equity in screening Aboriginal women.
Cairney and Abbott [29] Aboriginal wellbeing in a 'Red Dirt Economy'	Concept of health; Cultural practices; Identity; Working with community	Concept of health: Identified key themes of a well‐being framework: culture, employment, education, employment, health and spirit, community and mobility. Culture and empowerment were key factors in achieving positive well‐being outcomes, including learning skills and maintaining livelihood. Cultural practices: Education in culture can give healthy mind and heart; data from research shows clear and consistent link that culture is inseparable from relationships with Country and kinship, traditional language and passing on of traditional knowledge, skills and stories Identity: Researchers noted the need for work options that support Aboriginal people to maintain their own identity. The expectation to assimilate into mainstream ways of participating in workforce is at the cost of cultural identity Working with community: Research demonstrates necessity of engagement, collaboration and capacity development with Aboriginal and Torres Strait Islander peoples to achieve positive well‐being outcomes for remote communities	This study helped to build understanding of priorities for Aboriginal peoples—highlighting the importance of engagement, collaboration and capacity building to achieve positive outcomes
Jurgenson et al. [30], Exploring Australian Aboriginal women's experiences of menopause: a descriptive study	Concept of health; Working with community	Concept of health: Research findings reflect Aboriginal perspectives of changes at this time of life, with no clear consensus of how Aboriginal women refer to this transition, what language is used to describe this Working with community: Importance of acknowledging differences and similarities in experiences of menopause including use of language in conveying ideas and support for Aboriginal women	Developing a narrative about cultural and social influences on menopause. Considers Aboriginal perspectives of menopause, such as language, for example ‘change of life’
Lin et al. [31], ‘I can sit and talk to her’: Aboriginal people, chronic low back pain and healthcare practitioner communication	Cultural practices; Working with community	Cultural practices: Cultural communication practice of yarning as appropriate approach for future frameworks Working with community: Study describes miscommunication and enablers to good communication, language (terminology), personal experiences and engagement; Aboriginal people related better to health professionals when existing relationships to their community	Recommendations to health care practitioners about ways to improve communication to Aboriginal peoples about CLBP
Lopes et al. [32], Cross cultural education in suicide prevention: Development of a training resource for use in Central Australian Indigenous communities	Cultural practices; Working with community	Cultural practices: Applying principles of cultural safety within suicide prevention initiatives ensure they are more informed, more applicable and more effective Working with community: Resource developed through communication development and action research; concept of cultural safety recognises importance of localised and culturally specific approaches; power differentials between trainees and implementers emerged as concern; collaborative partnerships through development and implementation of resource	Culturally safe approaches to suicide prevention education increases trainees' knowledge and confidence. The aim of the programme is to reduce suicide rates in Aboriginal communities
Rae et al. [33], Long conversations: Gomeroi gaaynggal tackles renal disease in the Indigenous community	Concept of health; Cultural practices; Working with community	Concept of health: Health education programmes for Indigenous people need to address mind, body, spirit and communities Cultural practices: Local cultures need to be reflected ensuring cultural focus on aspects of health intervention Working with community: Sitting down sharing life stories establishes trust and builds relationships, programme developed alongside biomedical measures with community consultation, Elder's support and culturally safe space	Indigenous mothers, children, families and the Aboriginal health workforce benefited from this study. The programme improved cultural connection, family well‐being and health behaviours; strengthened cultural safety in care; supported workforce development; informed better education and prevention strategies for renal disease
Rix et al. [34], ‘Beats the alternative but it messes up your life’: Aboriginal people's experience of haemodialysis in rural Australia	Cultural practices; Working with community	Cultural practices: Identified vital role of family for the haemodialysis journey for Aboriginal people Working with community: Employing Aboriginal people within renal services to act as conduits between Aboriginal patients and hospital staff can bridge cultural gaps in services	Improved understanding of Aboriginal perspectives on dialysis and family support needs; enhanced cultural safety through patient‐led education for health professionals; and more culturally responsive care
Zander et al. [35], Indigenous Cultural and Natural Resources Management and Mobility in Arnhem Land, Northern Australia	Cultural practices; Identity; Working with community	Cultural practices: Investigated formal and informal engagement in cultural and natural resource management Identity: Land management practices impact looking after traditional country outside of formal work Working with community: Identified kinship rules in conducting research with certain participants and co‐researchers; adhering to cultural sensitivities when working with communities such as cultural taboos of co‐researchers and participants within community	Highlighted how paid involvement in formal land management (e.g., carbon farming, PES) supports Indigenous peoples to remain on Country, though it may also negatively influence traditional practices and mobility patterns.
Cuesta‐Briand et al. [36], Addressing unresolved tensions to build effective partnerships: lessons from an Aboriginal cancer support network	Cultural practices; Working with community	Cultural practices: Addresses cultural needs of the network peer support group Working with community: Results support the need to acknowledge and address different perspectives and world views in order to build strong, effective partnerships between service providers and Indigenous communities	Identified culturally appropriate ways to deliver support groups, highlighted the value of cultural brokers, and deepened understanding of working at the cultural interface between Aboriginal and mainstream services. Helped reveal differing values and approaches between Aboriginal peoples and service providers
Gladman et al. [37], Measuring organisational‐level Aboriginal cultural climate to tailor cultural safety strategies	Working with community	Working with community: Study provides insights about the perceived cultural climate of clinicians, academics and professional staff and how staff need to be aware of Aboriginal people's concepts of health and cultural needs to interact without prejudice	Improved cultural capabilities of clinicians, organisations and strengthened understanding of health as a holistic concept tied to cultural well‐being. Benefit to health staff working in Aboriginal health settings, and Aboriginal patients and clients
Helps and Barclay [38], Aboriginal women in rural Australia; a small study of infant feeding behaviour	Concept of health; Cultural practices	Concept of health: The study establishes Aboriginal model of view of health in context of breastfeeding Cultural practices: Knowledge and cultural norms are a shared resource within Aboriginal communities	This study explored barriers impacting breastfeeding among rural Aboriginal women to address rates of breastfeeding which could have deep, long lasting health benefits
Cairney et al. [39], Interplay well‐being framework: a collaborative methodology ‘bringing together stories and numbers’ to quantify Aboriginal cultural values in remote Australia	Concept of health; Cultural practices; Working with community	Concept of health: Development of a framework to explore concept and domains of Aboriginal health and well‐being through quantitative and qualitative methods Cultural practices: The model drew on survey domains such as language, Country, ceremony, family Working with community: Each component of the research conducted in shared spaces of stakeholders	Informed policy and practice through culturally grounded, community‐led research; strengthened capacity through training and employment of 42 local Aboriginal researchers; aligned government and community priorities; and advanced understanding of cultural determinants of wellness
McPhail‐Bell et al. [40], Deadly Choices empowering Indigenous Australians through social networking sites	Concept of health; Working with community	Concept of health: Indigenous health promotion, Deadly Choices uses SNSs as a tool for relationships to define and promote Indigenous health and healthy sense of Indigeneity and community Working with community: Using self‐determination and participants as health promotors in online discourse	The programme promoted health and well‐being through social networking sites (SNSs), which is a non‐traditional method of health promotion. It fostered online and offline community building and celebrated Indigenous culture and identity in digital spaces. It also repositioned Indigenous participants as health promoters and knowledge experts rather than consumers
Pilkington et al. [41], Perspectives of Aboriginal women on participation in mammographic screening: a step towards improving services	Concept of health; Working with community	Concept of health: Exploring factors impacting Aboriginal women's participation in breast screening Working with community: Screening programmes focus on inter‐cultural and inter‐sectorial partnerships with culturally appropriate programmes	Potential benefit to Aboriginal women by identifying key motivators for screening; for example investing in health for future generations and personal well‐being; emphasising the importance of peer support and social connections. Advocates for improved health literacy, education, and communication to enhance screening engagement.
Vujcich et al. [42], Yarning quiet ways: Aboriginal carers' views on talking to youth about sexuality and relationships	Concept of health; Cultural practices; Working with community	Concept of health: Young people's sexual health education in context of Family Empowerment Model, prioritising empowerment of carers to have conversations with young people about sexuality and relationships Cultural practices: Draw on cultural practices for interventions rather than mainstream Working with community: Role of culture and empowerment in improving relationships and sexual health outcomes	Highlights the need for interventions to support carers with the skills and confidence to deliver sexual health education. The study was of benefit to carers and young Aboriginal peoples to inform about barriers.
Schultz et al. [43], Indigenous land management (ILM) as primary health care: qualitative analysis from the Interplay research project in remote Australia	Concept of health; Cultural practices; Identity; Working with community	Concept of health: Explored ILM impacts of different domains of well‐being: health, empowerment, work, culture, education, community Cultural practices: ILM practices provide education for young people and transmission of knowledge Identity: ILM work recognises participant's identity and relationships with Country Working with community: Key stakeholders with previous research experience with the research centre were invited to participate	Illustrates how Indigenous Land Management (ILM) activities support well‐being—strengthening identity, relationships, and empowerment; promoting access to traditional foods and physical activity; limiting alcohol access; enhancing collaboration among community organisations. Advocates for greater involvement in ILM for holistic well‐being
Schultz et al. [44], Injury prevention through employment as a priority for well‐being among Aboriginal people in remote Australia	Concept of health; Identity; Working with community	Concept of health: Highlighting employment as important in Aboriginal well‐being Identity: Employment in Aboriginal ranger programmes strengthen sense of identity, which enhances well‐being Working with community: Shared space approach in collaborating between key stakeholders to prioritise community, culture and empowerment	Benefited Aboriginal communities by demonstrating how employment through a Ranger programme supports education and cross‐cultural learning, strengthens cultural identity, empowers women, reduces alcohol use, and provides meaningful opportunities for rehabilitation after prison
Schultz et al. [45], Re‐Imagining Indigenous Education for Health, Well‐being and Sustainable Development in Remote Australia	Concept of health; Cultural practices	Concept of health: Environmental health is related to Indigenous peoples' sense of well‐being Cultural practices: Participants established education for their children through knowledge transmission of cultural practices	The study benefited Indigenous children and communities showing that embedding Indigenous knowledge (art, culture, language, land management) supports children's well‐being, school attendance and literacy. Benefits for both Indigenous peoples and the nation
Armstrong et al. [46], ‘I've got to row the boat on my own, more or less’: Aboriginal Australian experiences of traumatic brain injury	Concept of health; Cultural practices; Identity	Concept of health: Explored individual stories of Aboriginal men faced with health issue highlighting cultural, psychological, geographical and service contexts Cultural practices: Provides understanding on holistic cultural views of health and beliefs about injury causes, cultural and systemic influence of recovery and service access are often overlooked Identity: Major identity changes due to impacts of oral communication due to injury	Benefited Aboriginal and non‐Aboriginal people who had a stroke or traumatic brain injury by improving understanding of challenges, recovery motivations and service gaps. Highlights the need for culturally informed care that recognises the importance of oral traditions, family roles and community connections for Aboriginal survivors
Dew et al. [47], Importance of Land, family and culture for a good life: Remote Aboriginal people with disability and carers	Concept of health; Cultural practices; Working with community	Concept of health: Gives voice to Indigenous Australian's with disability living in remote areas to understand their perception of good life and facilitating well‐being Cultural practices: Highlights the importance of engaging in cultural activities Working with community: Participants established ‘proper way’ for service providers to engage with Anangu perspectives to live a good life	Provides a strengths‐based understanding of what constitutes a ‘good life’ for Anangu peoples with disability. Provides evidence to support the development of culturally specific strategies to address challenges due to colonisation and geographic isolation
Schultz et al. [48], Australian Indigenous Land Management, Ecological Knowledge and Languages for Conservation	Concept of health; Cultural practices; Working with community	Concept of health: Explores how ILM contributes to well‐being of Aboriginal Peoples in remote regions Cultural practices: Ranger's participation in cultural activities promotes well‐being Working with community: Aboriginal peoples, researchers and government co‐developed a well‐being framework for the project	Employment in Indigenous Land Management (ILM) strengthens cultural connection through language and ecological knowledge, while also delivering clinical health benefits such as increased physical activity, improved nutrition, lower BMI, blood pressure, blood glucose levels, reduced stroke risk and less stress
Schultz et al. [49], Structural modelling of well‐being for Indigenous Australians: importance of mental health	Concept of health; Working with community	Concept of health: quantified the relationships between health care access, mental and physical health, and well‐being for well‐being for Indigenous Australians in remote regions Working with community: Enhanced health and well‐being for Aboriginal peoples through collaboration and strengthening collaboration of services beyond health sector, those that contribute to relationships, empowerment, cultural identity and Country	In context of remote communities' mental health links healthcare access to well‐being—informing the development of a validated survey for future use in Indigenous health research
Thompson et al. [50], Passing on Wisdom: exploring the end‐of‐life wishes of Aboriginal people from the Midwest of Western Australia	Concept of health; Working with community	Concept of health: Understanding Aboriginal perspectives and experiences at end‐of‐life Working with community: Discussions to engage local Aboriginal peoples to determine their end‐of‐life wishes	Provides broader insights into end‐of‐life issues for Aboriginal peoples. Educational video resources were produced for health staff. Recommendations were made to support more affordable, community‐led funeral options
Cox et al. [51], ‘It all comes back to community!’: A qualitative study of Aboriginal Elders promoting cultural well‐being	Concept of health; Cultural practices; Identity	Concept of health: Elders promoting cultural well‐being through mentoring, cultural healing and balancing mainstream health services and cultural foundations Cultural practices: Key role of Elders as teachers and interact with their community—promoting mentoring, cultural healing and advocating for cultural safety in health services Identity: Mentoring by Elders strengthens cultural identity and community connectedness, through maintaining connections to Country and community which are crucial to Aboriginal identity, and cultural and spiritual healing	Acknowledges Elders as promoters of cultural well‐being and community health. Highlights the diverse strategies Elders use to support well‐being and affirms their central role in sustaining cultural strength across generations

As expected, most studies were conducted outside of urban areas as publications were contributed to by UDRH researchers; Rural/Regional locations (*n* = 21); remote locations (*n* = 22); urban locations (*n* = 5). Studies were most commonly single‐site studies located in Western Australia (WA; *n* = 14), followed by the Northern Territory (NT; *n* = 4), New South Wales (NSW; *n* = 4) and Queensland (Qld; *n* = 1). Eight studies were conducted across multiple sites (NT and WA, *n* = 5; NT, SA and WA, *n* = 2). Two studies did not report the state where the research was conducted; one was undertaken online. Qualitative methods such as interviews or focus groups were most used for data collection.

Below we consider specific questions related to how the research of UDRH authors has approached and shone light on Aboriginal culture and health. Various themes emerged from collaborative yarning about how UDRH research has responded to Indigenous perspectives and priorities including self‐determined research, Indigenous leadership, governance and participation. We also considered ways in which Indigenous people as knowledge holders were respected and how findings from the research are communicated to Indigenous stakeholders and communities.

### 2.1 How UDRH Research Responded to Indigenous Perspectives and Priorities

Most papers (*n* = 27, 83%) included Indigenous peoples exclusively or part of their participant cohort. The remaining nine papers included both Indigenous and non‐Indigenous peoples in the study samples. However, 18 studies (54%) were classified as ‘not reported’ in terms of engagement with Indigenous peoples (Table 1). This engagement may come in the form of governance, community involvement and participant involvement, as found in [22] where Indigenous research assistants were employed, building their capabilities as researchers. Most (70%; *n* = 23) studies were also classified as ‘not reported’ when looking for information relating to feedback of findings to the community (where community refer to Indigenous participants, Indigenous community or organisations). A few studies described how participants were engaged throughout various stages of the studies but did not explicitly state or share in what ways the research findings were shared back to participants or community.

Some studies did state how research findings were disseminated to the community. For example, Passey et al. [24], shared research findings with the community reference group and [22] provided a summary of the research findings for each region. Other approaches were videos for educational purposes [50] and emailing findings to organisations and participants [28].

Only three papers mentioned participatory action research (PAR) processes in their studies [19, 29, 34]. Rix et al. [34] described PAR as a community‐based research process that aligns with IRM. The principles of PAR can enable researcher transparency and accountability and involvement of Indigenous peoples throughout the research process.

Self‐determined rural, remote and Indigenous health research activity is essential to improving research practices and health outcomes for Indigenous peoples and communities. Various studies detailed how the research came about and why it was initiated. For example, the studies of Nichols [19] and [43] with their colleagues described how their research was requested by Indigenous community leaders and the wider community due to ineffectiveness of current programmes. Numerous papers drew on existing literature to expose a health gap to justify the study, such as Coffin [22], Rix et al. [34], Lin et al. [25], Rix et al. [34].

In the 33 papers reviewed, a minority explicitly identified Indigenous authors and co‐researchers; others declared Indigenous community and Indigenous involvement in other areas of the papers such as in the methods, discussion or acknowledgment sections.

### 2.2 How UDRH Research Has Considered Indigenous Cultural Ways

The ‘Cultural Ways’ domains considered in the papers were: working with community (*n* = 27), followed by Indigenous concept of health (*n* = 24), cultural practices (*n* = 22) and identity (*n* = 10). Concepts about traditional healing (*n* = 1) and data sovereignty (*n* = 1) were rarely discussed in the studies. All papers considered at least one of the six domains of Indigenous HealthInfoNet's ‘Cultural Ways’ (summarised in Table 2), with many considering more than one domain.

Themes emerging from collaborative yarning about how UDRHs have given consideration to Indigenous cultural ways include: interactions between Western and Indigenous models of health, culture as a determinant of health, use of IRMs, involvement of Indigenous people in research processes from design, implementation and knowledge translation. Passey et al. [24] and Cairney et al. [44] highlighted the significance of drawing from Indigenous identity and culture to empower Indigenous involvement in research. This demonstrates that when Indigenous values are reflected in the research process, it results in culturally rigorous research.

There was an underwhelming number of papers which were aligned with categories of Traditional Healing (*n* = 1) and Data Sovereignty (*n* = 1). Overall, papers lacked explanation or mention of Aboriginal data sovereignty and collaborative rights of data ownership and usage. This is something that requires further attention to be included in future research. Specifically, UDRH‐led publications could do this given their collaborative approach as demonstrated in this narrative review.

Six (18%) of studies reported using yarning as a research method to collect qualitative data. Interviews were most common (*n* = 17); less commonly used were quantitative measures (*n* = 4 survey; *n* = 1 biomarker analysis). Interestingly, Lin et al. [25] described using a data collection method of interviews informed by yarning to ensure culturally inclusiveness of Indigenous communication methods. However, this also poses a paradoxical methodological approach at the cultural interface where an Indigenous research and communication method is being employed within a western research construct of ‘interviews’ as just a data collection method.

One of the Schultz et al. [48] studies to inform the development of a validated survey aimed to identify a quantitative measurement to reflect Indigenous well‐being from an Indigenous perspective. This may support future research designs to reflect Indigenous well‐being from an Indigenous lens, not retrofitted into existing western‐dominated health and well‐being models and frameworks.

### 2.3 Benefits Associated With UDRH Studies

Table 2 summarises the benefits associated with each study. Themes emerging from collaborative yarning about benefits associated with UDRH studies included: capacity building of Indigenous people in the research process, documenting experiences of health issues faced by Indigenous communities and informing culturally appropriate health programmes and research by giving voice to share Indigenous perspectives on health concerns and topics. Benefits of UDRH studies found in this review were the Indigenous involvement and governance in the UDRH research studies. This leads to building research capabilities of both Indigenous and non‐Indigenous peoples involved in research.

Most papers examined a health issue or concern which impacted the Indigenous community establishing perspectives and experiences with respect to dealing with that health phenomenon to be shared. Topics included breast feeding [38], breast cancer screening [41] chronic lower back pain (CLBP) [25] and experiences of wishes at end of life [50].

Studies shared ways that demonstrated cultural appropriateness of health promotion resources and sharing information cultural ways rather than individualised, biomedical approaches in clinical settings [20, 28, 32, 33, 39, 40]. Other studies explored the contemporary meanings attached to the use of bush medicine for Indigenous people accessing mainstream cancer treatment [21] and potential erosion of traditional cultural concepts of health if continued exposure to biomedically orientated approaches for CLBP [27].

## 3 Discussion

This historical analysis of UDRH Indigenous health‐related research provides insights of previous research and identifies opportunities for growth in Indigenous health. For this ‘deep dive’ into an analysis of the papers, authors privileged Indigenous perspectives to consider how UDRHs have considered Indigenous cultural ways in 12 years of research.

### 3.1 How UDRH Research Responded to Indigenous Perspectives and Priorities

#### 3.1.1 Indigenous Research Leadership

Self‐determined research should be a fundamental part of rural Indigenous health research [52]. Culturally safe research and Indigenous involvement should be self‐determined and involved in every step of the research process which cannot be rushed [52, 53]. Indigenous participation, such as co‐researchers and co‐investigators, grounded in meaningful, ongoing relationships and involvement from community, produces beneficial high‐quality research [53, 54]. Indigenous research leadership can come from Indigenous UDRH staff, Elders, local knowledge holders and Aboriginal community members.

Inclusion of Indigenous author cultural identity in scientific publications is emerging, providing context for the research. Stating Indigenous inclusion has not always been clear in research involving Indigenous peoples, making it difficult to assess Indigenous involvement and governance appropriately [1, 55]. Increasing Indigenous academic workforce and research collaborations will ensure that Indigenous authorship, involvement and governance across research processes occurs and is detailed appropriately in academic publications. By stating Indigenous authorship on UDRH academic publications, as is now required by some journals, this demonstrates to readers and Indigenous peoples and communities the involvement of Indigenous peoples in research projects which pertains to them.

#### 3.1.2 Knowledge Translation and Reporting Back to the Community

Lock et al. [56] describes how academic publications can exclude Indigenous experiences due to the non‐written communication methods of cultural knowledge. This may explain why aspects of knowledge sharing, feedback and translation were not always present in the papers reviewed, even though they may have occurred. Reasons for this include that they are done in other ways, are not reported in publications, and the limited space and word count of the journal. Moving forward, evidence of Indigenous involvement in all aspects of the research process is imperative to demonstrate rigorous, high quality Indigenous involvement and governance of health research.

While Indigenous communities may have positively experienced research in their community, what these benefits translate to is still contested [2, 57]. This narrative review provides a description of UDRH research relating to Indigenous health and culture and contributes to understanding of academic practices and outcomes. We must continue to explore and understand how Indigenous peoples and communities value and interpret the benefits of research outcomes [2]. Indigenous‐led UDRH research could explore Indigenous communities' perceived benefits and value of UDRH research and partnerships in their local areas.

Authentic co‐design is key to cultural governance and partnerships with Indigenous peoples as it centres Indigenous worldviews and knowledge, creating space for cultural authority and expertise to guide research [1, 58]. Studies in this review demonstrate co‐design within Indigenous health research contexts, geographically unique and challenging contexts, which empower local Indigenous peoples' involvement in all stages of the research, emphasising focus on knowledge translations and reporting back to the community.

Indigenous UDRH‐led research can lead the way in Indigenous rural and remote research by drawing on IRMs to enhance research process and experiences for Indigenous Australians and communities. CB‐PAR research and IRMs can further enhance UDRH research and collaborations. Indigenous health remains complex, requiring a broad set of research capabilities to overcome the knowledge gaps [59]. UDRHs continue to lead Indigenous rural and remote health research by drawing on community leadership and knowledge holders, developing trust between community and researchers [52]. This narrative review has demonstrated various ways to establish and uplift Indigenous research and voices in all stages of research processes.

#### 3.1.3 Leading Rural and Remote Indigenous Heath Research

This review shows what contributes to culturally safe academic spaces through culturally informed research collaboration and partnership with Indigenous researchers and communities. This review establishes the contributions of UDRH research on Indigenous health and culture to inform future research and health initiatives, to ensure they are culturally informed, contribute to equitable health service provision, and positive health outcomes for Indigenous peoples.

This narrative review examines historical research that embraced concepts related to Indigenous health and culture. We have highlighted Indigenous engagement and involvement to reflect research process and Indigenous voices and contributions [56]. Indigenous researchers and community partners provided expertise and demonstrated research leadership, resulting in successful partnerships with Indigenous communities.

Ongoing UDRHs investment is needed to enable and develop Indigenous research leadership, recognising the role of Indigenous academic researchers, knowledge holders, Elders and community members in informing research projects and agendas to respect local cultural knowledge, protocols and priorities. Ethical Indigenous engagement and involvement in research may prove difficult to reflect in one manuscript [54]. We encourage and advocate for Indigenous health research to ensure that this story is reflected in future publications to demonstrate the author's alignment with Indigenous research principles and demonstrate their ways of working with communities.

#### 3.1.4 Building Capabilities of Future Indigenous Researchers

Indigenous peoples play a vital role as cultural conduits between researchers and community. UDRH‐led research must continue to grow Indigenous health research leadership to ensure that the research outputs reflect cultural knowledge and context; in turn this contributes to the rigour of research outcomes and process [3]. Indigenous‐led research and authentic partnerships provide positive health outcomes through creating spaces for mutual learning, upskilling and capacity building [2, 53]. This review has identified how UDRH and Indigenous‐led research incorporates the cultural experiences and authority of Indigenous communities to ensure they are heard and established within academic literature.

UDRHs must continue to grow Indigenous health research leadership and workforce while simultaneously building the capability of non‐Indigenous researchers to ensure that Indigenous cultural values, protocols and worldviews are reflected and maintained in research spaces [7]. Indigenous peoples involved in research play key roles in shaping the research agenda and therefore the well‐being of Indigenous peoples [60].

Authors' cultural identity is increasing in publications which is particularly relevant for rural and remote health journals. Establishing cultural authority in Indigenous health research can help the reader understand Indigenous involvement and governance [56]. In the Contributors section of this paper, we recognise the cultural identity of Indigenous and non‐Indigenous authors, recognising the partnership and collaboration between Indigenous and non‐Indigenous UDRH staff. This helps with creating a culturally safe space for Indigenous involvement in western academic practices.

### 3.2 How UDRH Research Consideration of Indigenous Cultural Ways

#### 3.2.1 Culture as a Determinant of Health

Culture as a determinant of Indigenous health is well established within academic literature yet more research on this is warranted [55, 61]. This review reveals how UDRHs have contributed to understanding the cultural determinants of health as they relate to studies of Indigenous health and culture.

This current review recognises the role of culture in understanding certain health phenomena faced by Indigenous peoples across rural and remote Australia. It supports the growing body of work which identifies that if health programmes and research reflects Indigenous peoples' culture, with ethical and appropriate engagement, it will lead to greater support and sustainability by the Indigenous community. Interestingly, the [49] paper identifies and challenges issues with the current biomedical frameworks of health and the lack of reference to Indigenous people's well‐being priorities. This research demonstrates UDRH‐led research can utilise culture for Indigenous health programmes and research moving forward.

Papers in this review mainly focussed on descriptive research designs. Kennedy et al. [59] explains that descriptive research, on topics such as burden of disease, is required to inform future health programmes and publications and can provide understandings of the characteristics and determinants of Indigenous health. Continuing to grow Indigenous research capacity with a focus on cultural determinants of health can help move beyond a deficit‐descriptive statistical portrait of Indigenous health.

#### 3.2.2 Indigenous Research Methodologies

Brodie et al. [5] establishes that Indigenous research methodologies are increasingly informing culturally relevant research as historically Western methodologies have dominated research involving Indigenous communities. This review examined the presence of methodological approaches which align with Indigenous worldviews such as yarning, co‐design and CB‐PAR. Future research, particularly led by UDRH Indigenous staff and their colleagues, can embed Indigenous methodologies to empower self‐determined Indigenous research.

Yarning as an Indigenous research method privileges Indigenous voices and perspectives in all aspects of the research; however, the theoretical underpinning of utilising an Indigenous research method operating within Western research frameworks lacked explanation in this review [4, 14]. Indigenous research methods must be grounded and established within an overarching research paradigm to ensure Indigenous involvement and governance at all stages of the research. Yarning is more than just a data collection process; it is a way of knowing, being and doing across a research process, as demonstrated in the process of this review where authors (both Indigenous and non‐Indigenous) engaged with collaborative yarning to privilege Indigenous voices and elevate them [17]. Yarning must feature in all stages of Indigenous rural and remote health projects which aim to undertake research in a way that values Indigenous people, which are reflected in academic publications, beyond data collection.

Future UDRH research can re‐position Indigenous communities and peoples as health experts and advocates, recognising the role that Elders and communities play as health promoters of cultural well‐being and community health. This demonstrates the integral role that Indigenous academic researchers have, along with Elders and knowledge holders in communities throughout the research process. However, future UDRH Indigenous health research should aim for transparency and clearly identify Indigenous contributions and involvement in academic publications.

Overall, the benefits of individual studies vary as do the roles of Indigenous peoples and communities, from being research participants through to informing future policy and research scope. This analysis demonstrates the impact of UDRH‐led Indigenous research related to health and culture, highlighting the significance and vital role of culture and cultural health and well‐being in Indigenous health evaluation and research in rural and remote Australia.

#### 3.2.3 Aboriginal and Torres Strait Islander Quality Appraisal Tool [62]

The Aboriginal and Torres Strait Islander Quality Appraisal Tool (QAT) [62] has been developed from an Indigenous perspective to evaluate the quality of research involving Indigenous peoples. This validated tool is the first of its kind and it is generally viewed positively; however, it has been suggested that its application should be extended to support the research design phase to further promote ethical practices [63]. Quality assessments are not standard for narrative reviews, yet the tool did help inform our efforts on the data extraction reflected in Tables 1 and 2 [1]. In this review, we trialled the application of the QAT as an initial screening tool to assess the relevance and potential application of the included papers within the category. However, the group agreed that, given the publication dates of the papers which overwhelmingly preceded the more recent development and publication of QAT, it did not add value to apply the QAT across all studies given inconsistency in assessments by different assessors. Use of the tool does highlight deficits and strengths in Indigenous ethical research practices and will be a useful guide to support and strengthen future UDRH Indigenous research.

### 3.3 Limitations

As this narrative review is part of a larger project, we only considered those UDRH papers published between 2010 and 2021 coded as ‘Indigenous culture and health’. Thompson et al. [11], ensuring consistency with the extensive previous formative coding and analysis. Our findings were drawn from publications in the 12‐year period and are therefore historical in focus. We recognise there has been substantial progress in the paradigm and expectations for undertaking Indigenous research within the period under review and subsequently; changes not represented in this historical analysis. Coding processes prior to and within this analysis means some relevant UDRH studies may have been missed; however, our findings are likely to be robust with respect to the work undertaken during the period. The focus on individual papers in isolation from more extensive work with Indigenous people and communities by the same authors means that any single paper does not tell the full story around engagement, dissemination and translation. Hence, the information presented in the tables and results does not recognise the ongoing nature of relationship and engagement in research of UDRH researchers and their local Indigenous communities. The papers themselves show respectful and valuable contributions by UDRHs at local level, building Community Capital locally [10] and helping overcome some of the seagull approaches that have troubled much rural and remote Indigenous research [64].

## 4 Conclusions

This narrative review highlighted the studies included in the Indigenous health and culture category from the UDRH research and provides Indigenous perspectives and experiences of various health phenomena. This Indigenous‐led narrative review drew upon Indigenous research methods to guide the review process which centres Indigenous voices and experiences from the studies reviewed.

This study found that Indigenous‐led, collaborative and locally grounded research projects and processes contributed to successful outcomes for both academic researchers and Indigenous communities. Research that is Indigenous‐led and centres culture contributes to culturally safe and responsive rural and remote health research. The publications considered in this review reported various ways of Indigenous involvement in the research process, including co‐design and community researchers to contribute to culturally appropriate methods through recruitment, data analysis and knowledge translation. We must also continue to further explore how Indigenous peoples and communities value and interpret benefits of research outcomes [2].

Our review provides a description of UDRH research relating to Indigenous health and culture to contribute to understanding of academic practices and outcomes over a particular period. Various ways to establish and uplift Indigenous leadership and voices during research processes were demonstrated, recognising the key role of Indigenous people in the research and with Indigenous community partners. UDRHs continue to lead Indigenous rural and remote health research by drawing on community leadership and knowledge holders, which has been shown to develop trust between community and researchers [52].

Indigenous‐led UDRH research and collaborations informed by Indigenous peoples continue to push for changes in health and health systems to achieve positive health outcomes for Indigenous peoples and communities [59]. The review highlights that UDRHs need to demonstrate greater understanding and support for Indigenous data sovereignty in their Indigenous research. A better understanding of how UDRH research has incorporated elements of Indigenous cultural ways, the benefits of this research, and how the research has been conducted can improve future efforts in research with small rural and remote Indigenous populations. This narrative review intends to influence and encourage culturally safe and responsive rural and remote health research practices in partnerships with Indigenous researchers and communities.

## Author Contributions

**Michael Watkins:** conceptualization, data curation, formal analysis, methodology, project administration, visualization, writing – review and editing, writing – original draft. **Bahram Sangelaji:** data curation, writing – review and editing. **Ginger Minahan:** data curation, writing – review and editing. **Charmaine Green:** methodology, conceptualization, writing – review and editing, data curation, formal analysis. **Sandra C. Thompson:** conceptualization, data curation, formal analysis, writing – review and editing, writing – original draft. **Samantha Bay:** data curation, writing – review and editing. **Colleen Kelly:** conceptualization, methodology, data curation, formal analysis, writing – review and editing, writing – original draft.

## Funding

The authors have nothing to report.

## Conflicts of Interest

The authors declare no conflicts of interest.

## Data Availability

The authors have nothing to report.
